# Non-Canonical Inflammasome Pathway: The Role of Cell Death and Inflammation in Ehrlichiosis

**DOI:** 10.3390/cells12222597

**Published:** 2023-11-09

**Authors:** Aditya Kumar Sharma, Nahed Ismail

**Affiliations:** Department of Pathology, College of Medicine, University of Illinois at Chicago, Chicago, IL 60607, USA; aditya06@uic.edu

**Keywords:** *Ehrlichia* spp., non-canonical inflammasome, PAMPs, inflammasome, DAMPs

## Abstract

Activating inflammatory caspases and releasing pro-inflammatory mediators are two essential functions of inflammasomes which are triggered in response to pathogen-associated molecular patterns (PAMPs) or danger-associated molecular patterns (DAMPs). The canonical inflammasome pathway involves the activation of inflammasome and its downstream pathway via the adaptor ASC protein, which causes caspase 1 activation and, eventually, the cleavage of pro-IL-1b and pro-IL-18. The non-canonical inflammasome pathway is induced upon detecting cytosolic lipopolysaccharide (LPS) by NLRP3 inflammasome in Gram-negative bacteria. The activation of NLRP3 triggers the cleavage of murine caspase 11 (human caspase 4 or caspase 5), which results in the formation of pores (via gasdermin) to cause pyroptosis. *Ehrlichia* is an obligately intracellular bacterium which is responsible for causing human monocytic ehrlichiosis (HME), a potentially lethal disease similar to toxic shock syndrome and septic shock syndrome. Several studies have indicated that canonical and non-canonical inflammasome activation is a crucial pathogenic mechanism that induces dysregulated inflammation and host cellular death in the pathophysiology of HME. Mechanistically, the activation of canonical and non-canonical inflammasome pathways affected by virulent *Ehrlichia* infection is due to a block in autophagy. This review aims to explore the significance of non-canonical inflammasomes in ehrlichiosis, and how the pathways involving caspases (with the exception of caspase 1) contribute to the pathophysiology of severe and fatal ehrlichiosis. Improving our understanding of the non-canonical inflammatory pathway that cause cell death and inflammation in ehrlichiosis will help the advancement of innovative therapeutic, preventative, and diagnostic approaches to the treatment of ehrlichiosis.

## 1. Introduction

### 1.1. Canonical and Non Canonical Inflammasomes

PAMPs are microbial molecules that are detected by pattern recognition receptors (PRRs) such as toll-like receptors (TLRs) and various others that are part of innate immune system surveillance [1]. In addition to PAMPS, a wide variety of other molecules released by a cell during times of stress or infection, referred to as DAMPs (Danger-associated molecular patterns), are also sensed by PRRs [1]. One of the crucial PRRs of the innate immune system is inflammasomes, which control or trigger inflammation in response to PAMPs or DAMPs [1]. It has been demonstrated that inflammasomes are essential for the host’s ability to fight off infection [1]. However, the development of immunopathology, metabolic diseases, and neurodegenerative disorders have also been linked to dysregulated inflammasome activation [2,3]. Major inflammasomes include the NOD-like receptor (NLR) proteins, leucine-rich repeat (LRR)-containing (NLR) protein family members NLRP1, NLRP3, NLRC4, and proteins absent in melanoma 2 (AIM2) [4]. Some other less characterized inflammasomes are NLRP6, NLRP7, and NLRP12 [5]. Upon activation, these sensors initiate oligomerization of the adaptor protein ASC, which connects the upstream inflammasome sensor molecule to caspase 1 and leads to the generation of active caspase 1 [4]. Active caspase 1 triggers the cleavage of IL-1b and IL-18, in addition to the cleavage of Gasdermin D [4]. The cleaved Gasdermin D drives inflammatory cell death, known as pyroptosis, and leads to the release of biologically active IL-1b and IL-18 [6]. Although the major mechanism of inflammatory cell death in macrophages following infections with several Gram-negative and Gram-positive bacteria is dependent on gasdermin D [7], a recent study suggested that caspase 1/11 mediated the cleavage of gasdermin D, and in addition the subsequent pyroptosis, is cell specific. For example, the activation of both canonical and non-canonical inflammasome signaling pathways in neutrophils leads to Gasdermin D cleavage; however, this process was unrelated to cell death [8]. Unlike macrophages, gasdermin D cleavage in neutrophils following LPS stimulation is controlled by a neutrophil-specific serine protease and neutrophil elastase that is released from cytoplasmic granules into the cytoplasm [9]. The cleavage of gasdermin D in murine neutrophils can also occur via cathepsin G [10].

NF-κB is an important factor in the upregulation of NLRP3 sensors, following TLRs binding to PAMPs (or DAMPS), and the subsequent signaling via the adapter proteins MyD88, Toll-interleukin receptor domain-containing adapter protein inducing IFNβ (TRIF), or downstream kinases (IRAK1/4) [11,12]. NF-kB-mediated the activation of NLRP3 and is referred to as a classical/canonical pathway as it occurs via a two-step process [11]. The priming signal, or signal 1, causes an NF-kB mediated increase in levels of NLRP3 inflammasome components and post-translational changes to NLRP3 [11]. The second signal is mediated by the recognition of the different PAMPS and DAMPs as described below [11]. The PAMPs and DAMPs responsible for inflammasome activation and pathways are characterized in several models of infectious and non-infectious diseases [13,14]. NLRP1 responds to various PAMPs, such as an A/B toxin of *Bacillus anthracis* [13]. Rodents express three alleles of NLRP1, whereas humans express a single NLRP1 [15]. The NLRP1 pathway is also triggered by DAMPs associated with endoplasmic reticulum (ER) stress, which activates the inositol-requiring enzyme 1 α (IRE1α) and the protein kinase R-like ER kinase (PERK) [16]. AIM2 inflammasome is activated by microbial or host double-stranded DNA (dsDNA) [17]. Notably, AIM2 inflammasome activation following an infection with *Francisella novicida*, which resides within phagosomes, requires type I IFN (IFN-I), which upregulates IRF7 to induce GBPs and IRGB10. These GBPs lead to the opening of pores in the phagosomes and a release of bacterial ligands into the cytosol and the subsequent sensing by AIM inflammasomes [18]. NLRP3 is a notable inflammasome cytosolic sensor that is involved in several inflammatory diseases and is triggered by various ligands [2]. For NLRP3 inflammasome activation, there is no recognized unifying mechanism. It is not clear if NLRP3 is indirectly activated or activated by a specific ligand. Among the PAMPS and DAMPs that trigger NLRP3 activation directly (or indirectly) are pore-forming toxins, RNA-DNA hybrids, adenosine triphosphate (ATP), changes in intracellular Ca^2+^, lysosomal destabilization, oxidized mitochondrial DNA (mtDNA), mitochondrial reactive oxygen species (mtROS), and potassium efflux [19]. It has been suggested that potassium ion efflux is the universal overarching mechanism of NLRP3 activation [20]. Furthermore, it has been established recently that NIM-released kinase 7 (NEK7), which functions downstream of potassium efflux, is a crucial activator of NLRP3 in response to multiple stimuli [20].

Moreover, non-canonical activation of NLRP3 was recently described [11]. Within this pathway, lipopolysaccharides from the intracellular Gram-negative bacterial pathogens that reside within the membrane or phagosome enter the cytosol and trigger caspase 11 activation [11]. The guanylate-binding proteins (GBPs) and the immunity-related GTPase family member b10 (IRGB10) need to be produced in order to breakdown the bacterial membrane and enable the translocation of LPS, or other bacterium outer membrane molecules, to cytosol for it to be sensed by caspase-11 [21]. The LPS induced activation of caspase 11 leads to the activation of canonical NLRP3 inflammasome, likely via mechanisms that involve processing by pannexin 1 and potassium efflux [22]. The activation of NLRP3 inflammasome then triggers the cleavage of caspase 1 to process pro-IL-1β and pro-IL-18, which are subsequently released following gasdermin-induced pyroptosis [23]. Additionally, the activation of caspase 11 results in the production of the high-mobility group protein 1 (HMGB1) and IL-1α independently of caspase 1 [24].

The roles of caspases 1 and 11 in the endotoxemia caused by LPS was reviewed recently [6,25]. These studies indicated that caspase 11 primarily drives LPS-induced death, whereas caspase 1 induces the inflammatory response [25]. In another study, the activation of caspase 1 following the recognition of intracellular flagellin by NLRC4 causes the synthesis of eicosanoids, which are inflammatory mediators that drive inflammation and cell death without requiring IL-1β and IL-18 [26]. Nonetheless, studies employing infections with different pathogens suggest that the role of caspase 1 or caspase 11 in the pathogenesis of sepsis is pathogen-, tissue-, and/or organ-specific [13,24,26].

### 1.2. Non-LPS Mediated Activation of Non Canonical Inflammasome Pathway

As indicated above, the initial studies examining the non-canonical inflammasome pathways in sepsis models indicated that the LPS of Gram-negative bacteria are the most notable PAMPs driving this response. However, recent studies employing infections with protozoal, fungal, and viral infections suggested that non-LPS molecules are required for the activation of caspase 11/non canonical inflammasome pathways [27,28,29]. In an in vivo murine model of *leishmania*, lipophosphoglycan (LPG), a glycoconjugate, was identified as a molecule that activates caspase 11 [27]. However, in an in vitro culture, LPG fails to cause murine caspase 11 or human caspase 4 activation, suggesting that other microbial or host factors may be required for its action [27]. Other studies have shown that secreted aspartyl proteinases (Saps) of *Candida albicans* can also activate caspase 11 [28]. Sap2 and Sap6 triggers NLRP3 inflammasome activation canonically to secrete IL-1β and IL-18 [28]. The stimulation of murine macrophages with Sap2 and Sap6 increased caspase 11 activation, which was dependent on signaling via type I IFN receptors (IFNAR) [28]. In addition, studies have shown that outer membrane vesicles (OMVs) derived from *Bordetella pertussis* (BpOMVs) and transfected *B. pertussis* lipooligosaccharide (BpLOS) can also cause the activation of non-canonical inflammasomes and alter the cell-mediated immune response [29].

In addition to PAMPs, several DAMPs are also capable of caspase 11 activation [30,31]. For example, oxidized products of phospholipids, such as 1-palmitoyl-2-arachidonoyl-sn-glycerol-3-phosphorylcholine (oxPAPC), contribute to caspase 11 activation and elicit a pro-inflammatory response in dendritic cells specifically [30]. In models of Enterohemorrhagic *Escherichia coli* (EHEC) and *Citrobacter rodentium* infection, caspase 11 required TRIF signaling for its activation [31]. Mechanistically, the recognition of LPS in these Gram-negative bacteria by TLR4 recruits TRIF, which activates caspase 11 via the induction of IFN-I signaling [31]. Other studies suggested that ROS contributes to caspase 11 activation during infection with Gram-negative bacteria via the activation of RIP2 and JNK signaling [32]. Caspase 11 activation is also triggered upon the recognition of the lipoteichoic acid in Gram-positive *Staphylococcus aureus.* Notably, whereas several studies have suggested that caspase 11 plays a key role in a host’s defense against Gram-negative bacteria, a recent study has demonstrated that caspase 11 prevents the mtROS-mediated clearance of *S. aureus* in macrophages [33].

### 1.3. Negative Regulation of Inflammasomes

Inflammasomes are crucial for protecting against pathogens, whereas excessive activation can lead to systemic inflammatory disorders [2,14]. Several regulatory mechanisms that inhibit or prevent the activation of inflammasome pathways depend entirely on the tissues involved and the cellular context [13,24,34]. The regulation of inflammasomes occurs at the transcription and post-translation levels [35]. For example, epigenetic factors, such as DNA methylation and histone acetylation, control the NLRP3 transcriptional in mycobacterial infections [36,37]. MicroRNA is another known regulator of inflammasomes. The microRNA, miR-223, shapes the innate immune system during intestinal inflammation and regulates NLRP3 inflammasome in COVID-19 [38,39]. Other studies have demonstrated a direct relationship between miR-7 and NLRP3 in Parkinson’s disease pathogenesis, to which miR-7 triggers the microglial NLRP3 inflammasome activation [40,41]. In addition to miRNA, long non-coding RNA (lncRNA) Gm15441 targets the antisense transcript of the thioredoxin-interacting protein (TXNIP) that suppresses the TXNIP activation of oxidative stress induced-inflammasome activation [42]. Thus, targeting miRNA- and lncRNA could be a potent approach for the treatment of conditions associated with NLRP3 dysregulation and excessive inflammation [38,42].

At the post transcription level, nitric oxide produced as a result of the host’s response during *Mycobacterium tuberculosis* infections inhibits NLRP3 inflammasome activation via thiol nitrosylation [35]. Nitric oxide also negatively regulates inflammasome by stabilizing mitochondria and decreasing mtROS [35,43]. Similarly, anti-inflammatory cytokine IL10 reduces mtROS production, inhibiting the NLRP3 inflammasome activation [44]. Additionally, the inhibition of K^+^ efflux by ketone bodies, specifically β-hydroxybutyrate (BHB), can inhibit the NLRP3 inflammasome [45]. Other signaling pathways that negatively regulate inflammasome include WNT/β-catenin signaling pathways, which are involved in liver regeneration and growth [46]. Heat shock transcription factor 1 interaction with β-catenin controls the activation of XBP1 to regulate the NLRP3 inflammasome in murine models of hepatic ischemia/reperfusion injury [46]. In addition, the IRE1α signaling pathway, which is one of the three arms of the unfolded protein response (UPR) inhibits the NLRP3 assembly, and subsequently, caspase 1 activation [47]. Thus, understanding these negative regulations could enable the development of novel approaches for targeting inflammasome under conditions in which immunopathology and excessive inflammation following inflammasome activation are deleterious.

### 1.4. Positive Regulation of Inflammasome-Mediated Inflammatory Cell Death by Caspase 3, 7, and 8

Cell death occurs via either the mitochondrial apoptotic pathway (intrinsic) or the death receptor pathway (extrinsic) [48]. In the mitochondrial apoptotic pathway, cytochrome c (cyt c) is released from the outer mitochondrial membrane, which leads to the formation of an apoptosome which activates caspase 9 [48]. Active caspase 9 then activates caspase 3, which mediates the apoptotic cell death [48]. Recent studies suggested that caspase 3 mediates apoptosis and is followed by pyroptotic cell death by the cleavage of Gasdermin E (GSDME), which plays a crucial role in non-canonical mediated pyroptosis [49]. It has been observed that GSDME is cleaved by caspase 3 to induce pyroptosis during viral infection [50] and in tumors treated with chemotherapeutic drugs or following stimulation by tumor necrosis factor (TNF) [50]. Similarly, following pulmonary infections with Gram-positive bacteria such as *streptococcus pneumonia*, the ability of caspase 3 to shift cell death from apoptosis to pyroptosis has been associated with the development of pneumonia and lung injury [51]. These studies suggest that GSDME cleavage by apoptotic caspase 3 is induced to regulate the disassembly and progression of apoptosis to pyroptosis [51]. Additionally, GSDME expression dictates the cell death mechanism, to which increased expression of GSDME shifts the cells from apoptosis to pyroptosis [49]. This conclusion is based on the findings that tumor cells have very little or no expression of GSDME, and that the increased expression of GSDME upon treatment with chemotherapeutic drugs triggers pyroptosis in tumor cells [52].

Caspase 7 is another apoptotic effector; however, caspase 7 is inefficient in driving apoptosis alone [53]. Recently, it has been shown that caspase 1 activates caspase 7 during infections with pathogens such as *Salmonella typhimurium*, *Chromobacterium violeceum*, and *Listeria monocytogenes* infection models, to which caspase 7 activation protects against cellular injury by driving the plasma membrane repair mechanisms [54,55]. In response to *S. typhimurium* infection, GSDMD forms pores in the plasma membrane, which is predominantly a non-canonical mediated pyroptotic feature [56]. These pores release caspase 7 and acid sphingomyelinase (ASM) into the extracellular space, which exits the cell to interact with, and be cleaved by, caspase 7. The cleaved ASM removes the head group of sphingomyelins, a significant component of animal cell membranes, to generate ceramide [56]. Ceramide repairs the plasma membrane by inducing endocytosis in the GSDMD pores, thus delaying cell lysis so it may carry out the extrusion of intestinal epithelial cells (IEC) in *S. typhimurium* infections [56]. A similar process occurs in response to *C. violeceum* and *L. monocytogenes*, in which the host’s protective tactics are used to block cell death and facilitate cell survival in response to pore formation via gasdermin D [54,55]. The exact mechanism of whether caspase 1 directly activates caspase 7 or whether it activates through an intermediate caspase, namely caspase 11, remains elusive.

Among the apoptotic caspases, caspase 8 is categorized as an initiator caspase that activates effector caspases (caspase 3/6/7) following extrinsic signals, resulting in apoptosis [57]. Caspase 8 is activated through an extrinsic pathway following the binding of TNF-to-TNF receptors. Caspase 8 can also activate an intrinsic pathway, which involves the cleavage of Bid, a member of a cytosolic B-cell lymphoma consisting of two family members [58]. Recent studies have shown that caspase 8 plays a role in inflammation through the cleavage of GSDMD, which drives pyroptosis and other pro-inflammatory processes, including NF-κB activation, cytokine production, and autophagy [58]. Caspase 8 cleaves GSDMD either directly or through the caspase 8-mediated activation of caspase-1 [59,60]. However, caspase 8 triggers the activation of the non-canonical NLRP2-ASC-caspase 8 inflammasome. This results in the death of dendritic cells in a manner that is independent to caspase-1 during infections with fungal pathogens such as *Cryptococcus neoformans* [61]. Together, these studies highlight the importance of caspase 3 and caspase 8 in the roles they play promoting apoptotic and pyroptotic cell death following the activation of canonical and non-canonical inflammasome pathways.

Furthermore, researchers have recently identified a new form of coordinated cell death pathways called PANoptosis, which is regulated by the multiprotein complex PANoptosome [62]. This programmed cell death pathway has three components: pyroptosis, apoptosis, and necroptosis, which are well coordinated and regulated [62]. Additionally, studies have shown that ZBP1, an innate immune sensor, can sense viral RNA products and endogenous nucleic acid to induce PANoptosis [63]. When ZBP1 senses these stimuli, it recruits RIPK3 and caspase-8 to activate ZBP1-NLRP3 inflammasome, in addition to other host factors involved in type I interferon and caspase 6 signaling, which are essential for ZBP-1-NLRP3 inflammasome assembly [63]. PANoptosis is a relatively new area of research, however there is the potential for it to result in new therapeutic strategies for a variety of diseases.

## 2. Human Monocytotropic Ehrlichiosis (HME)

### 2.1. Clinical Presentations

HME is a tick-transmitted, zoonotic, potentially fatal infectious disease primarily caused by obligately intracellular bacteria, *Ehrlichia chaffeensis* [64,65,66,67]. According to the US Centers for Disease Control and Prevention (CDC), the cases of ehrlichiosis have increased tenfold from 2001 to 2019 [65]. However, the cases of HME are underestimated due to the lack of specific clinical manifestations, non-specific laboratory findings, and a lack of high sensitivity and specificity diagnostic testing [65]. Patients with HME can present with a mild, non-specific, flu-like illness that is associated with an elevated level of liver enzymes and thrombocytopenia [65]. Other patients can present with severe and potentially fatal diseases and associated complications such as aseptic meningitis, adult respiratory distress syndrome, toxic shock, and multi-organ failure [64,68,69]. The liver pathology, which is characterized by apoptotic hepatocellular cell death, the activation of monocytes and tissue-resident macrophages, multifocal inflammatory lesions, and steatosis, are major pathognomonic findings in HME patient’s liver biopsies [65,70,71]. Doxycycline is the drug of choice for treating HME; however, late treatment fails to prevent the development of severe disease. HME is a major health problem due to a high hospitalization rate of 53% to 72%, and a mortality rate of approximately 2% [64,65]. HME is more frequently encountered in men than women and in older patients between the ages of 60–69 [65]. Individuals with weakened immune systems, such as organ transplant or HIV patients, are more susceptible to infection in addition to the development of severe and potentially fatal outcomes [64,65].

### 2.2. Pathogenesis of HME and Immunopathology

*E. chaffeensis*, the most notable etiology of human ehrlichiosis in the US, followed by *E. Ewingii,* are members of the Anaplasmataceae family in the order of Rickettsiales [72,73,74,75]. *Ehrlichia* species are first considered a veterinary pathogen that can infect many animal hosts. Humans can contract potentially fatal or severely debilitating diseases as a result of transmission and infections in domesticated animals. The major *Ehrlichia* species that cause HME are: *E. chaffeensis*, *E. ewingii, E. canis*, and *E. muris subsp. eauclairensis* (previously known as an Ehrlichia muris-like agent [72,75,76,77,78,79]). *Ehrlichia* sp. HF565, isolated from *Ixodes ovatus* ticks in Japan (and known as *Ixodes ovatus Ehrlichia* (IOE)/*E. japonica*), causes an acute and fatal infection in mice in the laboratory and matches the pathological, immunological, and clinical findings in patients with severe and potentially fatal HME [65,80]. The most notable target cells for the Ehrlichia species are monocytes and macrophages [65]. *Ehrlichia * also known to infect endothelial cells and hepatocytes, and such infections were found to result in liver injury and hepatocellular cell death [65,71].

Cell-mediated immunity contributed significantly to the host’s defense against *Ehrlichia* infection [81]. During *Ehrlichia* infection, there is intricate equilibrium between protective and pathogenic immune responses that determine the course of the disease [82]. For instance, CD4 Th-1 and NKT cells are known to generate IFN-γ, which triggers the macrophages with microbicidal abilities that help to clear *Ehrlichia* and provide a protective response. However, these CD4 Th-1 and NKT cells die during the later stages of severe *Ehrlichia* infection, which results in more severe HME response. Whereas CD4 Th1 and NKT cells play a protective role, TNF-a producing and cytotoxic CD8^+^ T cells or NK cells play a pathogenic role during fatal ehrlichiosis [83]. Furthermore, our research has demonstrated that neutrophil depletion attenuated liver damage and the expansion of pathogenic CD8^+^ T cells, in addition to a promoted resistance to fatal ehrlichiosis, suggests that neutrophils also contribute to a host’s susceptibility to a fatal infection [81,84]. Notably, studies from our lab have found that the secretion of inflammasome-dependent cytokines such as IL-18 contribute to the development of fatal ehrlichiosis by promoting the induction and expansion of pathogenic CD8+ T cells and NK cells. Employing the murine model of fatal ehrlichiosis, we found that mice deficient in IL-18 receptor (IL-18R^−/−^) had a prolonged survival following IOE infection compared to wild type mice [85]. Mechanistically, a lack of IL-18R signaling in IOE-infected mice decreased the expansion of pathogenic CD8^+^ T cells, which led to the restoration of the protective CD4^+^ Th1 response, a decrease in inflammation, and attenuated tissue damage [85]. These studies showed that during *Ehrlichia*-induced sepsis in mice, inflammasome is essential for the activation of pathogenic innate and adaptive immune responses, and these will be discussed in detail in the next sections.

## 3. Role of Canonical and Non-Canonical Inflammasome Pathway in Ehrlichiosis

### 3.1. Canonical Inflammasome Pathway (s) in Ehrlichiosis

Our research has shown that canonical and non-canonical inflammasomes get activated in ehrlichiosis [83,86,87]. The increased expression of inflammasomes, such as NLRP3, NLRP1, NLRC4, NLRP12, and AIM2, and caspase 1 and caspase 11 activation has been linked with fatal ehrlichiosis in mice [86,88,89]. Mice deficient in caspase 1 and infected with virulent IOE died from an infection early in comparison to wild-type mice [89]. Caspase 1 deficient mice were less effective at clearing the *Ehrlichia* infection and developed extensive liver injury compared to wild type mice and mice deficient in NLRP3 [89]. These data suggest that NLRP3 activation via non-canonical pathways is a key mediator of immunopathology following lethal *Ehrlichia* infection. However, the enhanced susceptibility of caspase 1 deficient mice to fatal ehrlichiosis indicates that caspase 1 plays a protective role in the effective antimicrobial host’s defense against *Ehrlichia* [89]. This antimicrobial effect of caspase 1 could be due to caspase 1-mediated pyroptosis. In infections with *Salmonella* and *Burkholderia* species, caspase 1 induced pyroptotic cell death in addition to the clearance of bacteria by reactive oxygen species in neutrophils, and moreover, this occurred without the release of IL-1b and IL-18 [90]. Lethal *Ehrlichia* infection induces the activation of neutrophils and their migration to the site of the infection including the liver [81]. Thus, it is possible that caspase 1-induced pyroptosis leads to a release of intracellular *Ehrlichia* from the infected macrophages, the main target cells. These extracellular bacteria can then be phagocytosed by activated neutrophils and killed via ROS, as suggested in other infection systems [91]. As described above, neutrophils are resistant to Gasdermin-mediated pyroptosis in response to certain inflammasome activators. Thus, the link between caspase 1 activation at the sites of infection and the subsequent killing by neutrophils may account for the host-protective function of caspase 1.

Apart from the potential antimicrobial effect of caspase 1, the finding that caspase 1^−/−^ mice develop extensive liver damage following *Ehrlichia* infection suggests that caspase 1 is likely hepatoprotective [81]. This conclusion is supported by studies showing that caspase 1^−/−^ deficiency mice developed systemic inflammation and liver damage in a hemorrhagic shock model [81]. Interestingly, patients who survive sepsis have increased expression of caspase 1, which correlates with a decreased expression of caspase 3 [92]. Similarly, macrophages lacking caspase 1 that are infected with *Francisella* express elevated levels of caspase 3 and undergo apoptosis, suggesting a causal link between caspase 1 and caspase 3 [93]. As we detected an inverse relationship between the caspase 1 and caspase 3 expressions, we examined whether the potential hepatoprotective role of caspase 1 is due to the inhibition of caspase 3.

### 3.2. Non-Canonical Inflammasome Pathways and Their Regulation by Type I- IFN in Fatal Ehrlichiosis

Unlike caspase 1, we have found that non-canonical inflammasome signaling is likely a pivotal inducer of liver injury during severe and fatal ehrlichiosis [86,87]. Furthermore, we have showed that caspase 11 activation in fatal ehrlichiosis is regulated by type I interferon (IFN-I), and that IFN-I mediated caspase 11 activation accounts for immunopathology and fatal outcomes following infection with virulent IOE [87]. In comparison to wild type mice, IFN-I receptor deficient mice were resistant to fatal *Ehrlichia* infection [94]. IFNAR-I deficient mice had a significant reduction in the activation of caspase 11, and moreover, the production of IL-1b, which highlights the importance of IFN-I mediated regulation of non-canonical inflammasomes in fatal ehrlichiosis [94]. Using murine bone marrow chimera, it was found that the expression of IFN-I receptor (IFNAR) on non-hematopoietic cells during fatal *Ehrlichia* infection is essential for a fatal outcome following IOE infection. Recently, we demonstrated that virulent IOE infects primary murine hepatocytes and that IFNAR signaling on hepatocytes promotes bacterial replication and positively regulates caspase 11 activation and inflammation [87]. IFNAR-mediated regulation of caspase 11 activation in hepatocytes resulted in the secretion of IL-1b, IL-1a, and HMGB1, as well as pyroptosis/inflammatory cell death [87]. In addition to IFNAR-mediated positive regulation of non-canonical inflammasome during fatal IOE infection, IFNAR signaling also results in the loss of bone marrow and a reduction of hematopoietic stem and progenitor cells (HSC/HSPCs) via lower expression and activity of caspase 8 [95]. The latter prevents the cleavage of RIPK1 and leads to RIPK1-kinase-dependent cell death [95].

How IFN-I regulates the non-canonical inflammasome pathways during *Ehrlichia* infection remains elusive. IFNAR can promote non-canonical NLRP3 inflammasome activation by increasing the abundance of the caspase 11 protein [83]. Alternatively, as suggested in other studies, infection with Gram-negative bacteria triggers an IFN-I response, which results in the increased expression of genes that encode guanylate binding proteins (GBPs) [18]. GBPs promotes bacterial lysis to release cell wall or bacterial components, which can lead to the activation of non-canonical NLRP3 inflammasome pathways [83]. In addition, GBPs open pores in the phagosomes in which bacteria reside, enabling access for bacterial LPS from the phagosome into the cytoplasm and the subsequent activation of caspase 11 [83]. As *Ehrlichia* lack LPS, there is the possibility for other PAMPs to be released into the cytosol, that may lead to the activation of IFNAR signaling. Recent research showed that mice lacking Caspase 11 can survive longer than wild-type mice when infected with SARS-CoV-2, indicating that targeting this pathway could be a promising therapeutic approach [96].

### 3.3. Potential PAMPS and DAMPS That Trigger Inflammasome (s) Activation in Ehrlichiosis

As *Ehrlichia* lacks LPS as well as peptidoglycan, it is less likely that LPS-like molecules can trigger the IFN-I-caspase11 axis. However, other *Ehrlichia* PAMPS may trigger inflammasome activation in ehrlichiosis. Like other intracellular bacteria, *Ehrlichia* utilizes the type IV secretion system (T4SS) to secrete proteins that might be PAMPs or toxins, which access the cytosol and may activate the immune system via activating inflammasome signaling [97]. Studies have shown that *Helicobacter pylori* secrete CagA (Cytotoxin-associated gene A) into the epithelial cells to activate the NLRP3/caspase 1 axis to secrete IL-1β, which hastens inflammation in atherosclerosis [98]. Similarly, *Legionella pneumophila*, an intracellular bacterium that is known to exploit the inflammasome pathway, especially non-canonical inflammasomes, employ a type IV secretion system (T4SS) that has been shown to activate caspase 3 using several T4SS substrates, including VipD, the phospholipase A2 that destabilized the outer mitochondria membrane to free cytochrome c and subsequently triggered caspase 3 [99,100,101]. *E. chaffeensis* translocated factor 1 (Etf1), *E. chaffeensis* translocated factor 2 (Etf-2), and *E. chaffeensis* translocated factor 3 (Etf-3) are a small number of T4SS effector proteins secreted abundantly during *Ehrlichia* infection [102]. Etf-3 is shown to induce ferritinophagy in the host cell, which leads to an increase in labile cellular iron in the host cell, which can activate the inflammasome [102]. However, recent studies have identified the ankyrin domain’s role in stabilizing active caspase 1 [103]. Therefore, it might be possible that the T4SS effectors function as a critical inflammasome adaptor that fine tunes the activation of different caspases during *Ehrlichia* infection. Despite lacking the genes necessary for the synthesis of cholesterol in their cell walls, *Ehrlichia* hijacks the host cells and relies on the phospholipids in their cell wall for survival and infection [65,102]. These exported phospholipids and cholesterol may act as *Ehrlichia* PAMPS that elicit inflammasome activation.

Other *Ehrlichia* PAMPS that access cytosol and could trigger the activation of non-canonical inflammasome pathways are tandem repeat proteins (TRPs) [104]. TRPs are immunomodulin proteins, type I secretion effectors (T1SS), that are present at the ehrlichial surface or secreted in extracellular space [104]. TRP32, TRP47, TPR75, and TRP120 are known TRPs found in *Ehrlichia* [104]. These TRPs interact with diverse host proteins and modulate several cellular signaling events [104]. TRP32 can also control apoptotic function during *Ehrlichia* infection by interacting with GLCCI1 and TP53I11, and through its interaction with CD14, TRP32 influences MAPK, TLR, and IKK/NFκB signaling [104]. Similarly, TRP47 interacts with adenylate cyclase-associated protein 1 (CAP1) to alter its mitochondrial shuttling to promote apoptosis [104,105]. TRP47 also translocates to the nucleus using MYND-binding proteins to interact with genes that regulate actin cytoskeleton organization and immune response [106,107]. *Ehrlichia* also secretes TRP75, a predicted lipoprotein, which has been shown to interact with MMP9 (Matrix metalloproteinase-9), which has a role in cytokine-mediated signaling [104]. TRP75 can alter cellular metabolism by binding with protein kinase AMP-activated catalytic subunit alpha 1 (PRKAA1), which is the catalytic subunit of the AMP-activated protein kinase (AMPK) that promotes autophagy during energy stress [104]. Furthermore, the *E. chaffeensis* TRP120 effector protein exploits the host SUMOylation pathways for their intracellular survival [108]. In addition, TRP120 function as a nucleomodulin, which binds to GC-rich regions of host DNA to regulate multiple cellular functions to promote *Ehrlichia* infection positively [109,110]. Canonical Notch signaling is also triggered by *E. chaffeensis* TRP120 to decrease TLR2/4 expression and increase survival within the cells [111].

In addition to *Ehrlichia* PAMPs, several potential DAMPs generated during infection with virulent *Ehrlichia* are likely triggering activation of inflammasome [65]. Infection of macrophages with virulent IOE resulted in inhibition of autophagy induction and flux via MyD88 signaling, which result in faulty mitophagy and build-up of damaged mitochondria and ROS [86]. Consequently, the mtDNA or mtROS are likely to function as DAMPS activating NLRP3 inflammasome [86].

## 4. Conclusions

**Figure 1:**
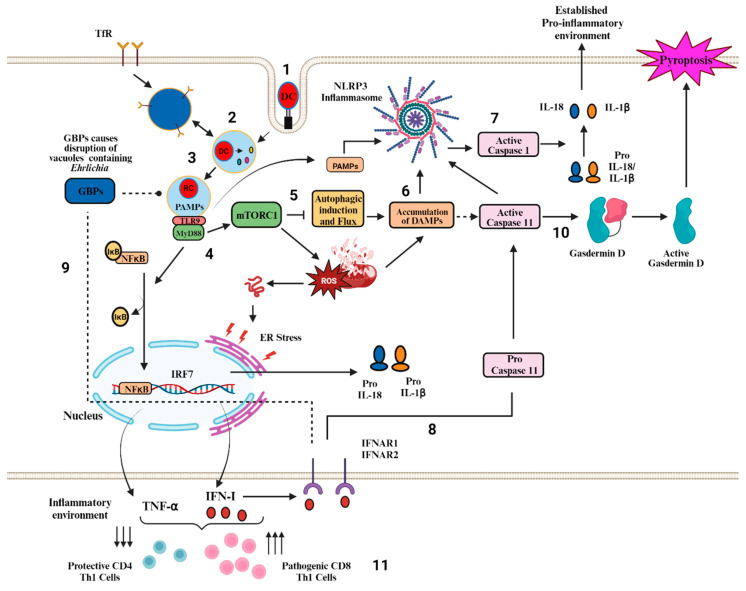
Model of Activation of canonical and non-canonical Inflammasome Pathways during severe and potentially fatal *Ehrlichia* infection. (**1**) Dense core cells enter macrophages. (**2**) Bacteria replicate within an *Ehrlichia*-containing vacuole (ECV) resembling early endosomes, secreting type I and type IV secretion system effectors including TRP32, TRP47, TRP120, and Ank200. (**3**) Dense core cells differentiate into reticulate cells and fuse with TfR endosomes to acquire iron and disrupt host cell signaling. (**4**) *Ehrlichia* PAMPS or DAMPS generated in infected cells such as mitochondrial DAMPS induces TLR9/MYD88 downstream targets, activating the NF-κB pathway. Activated NF-κB acts as a transcription factor to upregulate NLRP3, TNF-α, pro-caspase 1/11, pro-IL-1β, and pro-IL-18 providing the first signal required for the activation of canonical inflammasome pathway. (**5**) MYD88 signals in infected macrophages also cause a block of autophagy induction via mTORC1 activation as well as blocking autophagy flux causing defective mitophagy and mitochondrial damage/dysfunction and ER stress. (**6**) MyD88 mediated block of autophagy flux triggers an accumulation of DAMPS such as mtDNA or ROS. (**7**) Canonical NLRP3 inflammasome activation occurs via unknown mechanisms, but it may involve recognition of DAMPs and/or *Ehrlichia* PAMPs secreted to the cytosol via type I and type IV secretion systems. NLRP3 signaling leads to Caspase 1 activation and the subsequent cleavage of pro-IL1β and pro-IL-18 into their mature forms. (**8**) *Ehrlichia* also triggers the upregulation of IRF7 and the production of Type I IFN cytokines, which signal via the type I IFN receptor (IFNAR) and positively regulate activation of non-canonical inflammasome pathways leading to Caspase 11 activation. (**9**) The PAMPs that trigger Caspase 11 activation during infection with LPS negative *Ehrlichia* is unknown, but it is possible that IFN-1 and IFNAR signaling induce GBPs, which may disrupt *Ehrlichia*-containing vesicles, releasing PAMPs to the cytosol. (**10**) Activation of the non-canonical inflammasome pathway marked by Caspase 11 activation leads to the cleavage of Gasdermin D and a release of IL-1β and IL-18 in addition to pyroptosis of the host cells causing tissue damage. (**11**) Excessive inflammation secondary to inflammasome activation, together with inflammatory cell death promotes the expansion of pathogenic cytotoxic CD8+ T cells, causing further cytotoxic cell death and extensive tissue damage. The MYD88-induced block of autophagy may negatively impact MHC-II antigen presentation and may lead to the attenuated activation and proliferation of protective CD4+ Th1 cells, which in turn promote bacterial replication and dissemination. (Biorender.com, (accessed on 1 October 2023)).

Recent work by our lab and others has deciphered the *Ehrlichia* interactions with the host in in vivo and in vitro models. Using two strains, *E. muris* and IOE/*E. japonica* for the infection in mice, valuable insights have been drawn regarding the mechanisms that govern protection and immunopathogenesis. The *Ehrlichia*-host interaction is multifaceted in nature, in which many signaling mechanisms and pathways play an essential part (Figure 1). The emerging role of non-canonical inflammasomes adds complexity to understanding the immune response triggered by *Ehrlichia*. For ehrlichiosis, no other drugs are available except for doxycycline, which is for early treatment of ehrlichiosis. In addition, no vaccine is available for ehrlichiosis. However, as we continue to unravel the molecular intricacies of the non-canonical inflammasome pathways that lead to cell death and inflammation, these new insights hold promise for developing targeted therapeutics for treating ehrlichiosis during the later stages of the infection. In conclusion, by shedding light on these mechanisms, this review paves the way for further investigations of non-canonical inflammasomes and ehrlichiosis.

## Data Availability

Not applicable.
